# The impact of maintaining serum potassium ≥3.6 mEq/L vs ≥4.5 mEq/L on the incidence of new-onset atrial fibrillation in the first 120 hours after isolated elective coronary artery bypass grafting – study protocol for a randomised feasibility trial for the proposed Tight K randomized non-inferiority trial

**DOI:** 10.1186/s13063-017-2349-x

**Published:** 2017-12-28

**Authors:** Niall G. Campbell, Elizabeth Allen, Julie Sanders, Rebecca Swinson, Sophie Birch, Joanna Sturgess, Nawaf Al-Subaie, Diana Elbourne, Hugh Montgomery, Ben O’Brien

**Affiliations:** 1 Department of Cardiology, Wythenshawe Hospital, Manchester University Foundation NHS Trust, Southmoor Road, Manchester, M23 9LT UK; 2 Clinical Trials Unit, London School of Hygiene & Tropical Medicine, Keppel Street, London, WC1E 7HT UK; 3 Barts Heart Centre, St Bartholomew’s Hospital, Barts Health NHS Trust, West Smithfield, London, EC1A 7BE UK; 4 Cardiothoracic Intensive Care Unit, St George’s Hospital, Blackshaw Road, Tooting, London, SW17 0QT UK; 5 UCL Division of Medicine, and Institute for Sport, Exercise and Health, 1st floor, 170 Tottenham Court Road, London, W1T 7HA UK; 6 William Harvey Research Institute, Queen Mary University of London, Charterhouse Square, London, EC1M 6BQ UK; 7 Outcomes Research Consortium, Cleveland, OH USA

**Keywords:** Potassium, Coronary Artery Bypass Grafting, Atrial Fibrillation, Cardiac Surgery

## Abstract

**Background:**

Atrial fibrillation (AF) occurs in approximately one in three patients after cardiac surgery, and is associated with increased short-term and long-term mortality, intensive care unit (ICU) and hospital stay, and increased cost of care. In an attempt to reduce AF incidence in these patients, serum potassium (K+) levels are commonly maintained at the high end of normal (4.5–5.5 mEq/L). However, such potassium supplementation is without proven benefit, and is not without negative consequences. It carries clinical risk, negatively impacts patient experience and is both time-consuming and costly. This protocol describes a randomised controlled pilot trial to assess the feasibility of a proposed randomised non-inferiority trial to investigate the impact of maintaining serum potassium ≥ 3.6 mEq/L vs ≥ 4.5 mEq/L on the incidence of new-onset atrial fibrillation in the first 120 hours after isolated elective coronary artery bypass grafting.

**Methods:**

Design: this is a randomized feasibility trial as a pilot for a randomized non-inferiority trial. Participants: are 160 patients undergoing isolated coronary artery bypass grafting at two centres. Allocation: patients will be randomized (1:1) to protocols aiming to maintain serum potassium at either ≥ 3.6 mEq/L (“relaxed control”) or ≥ 4.5 mEq/L (“tight control”). Primary analytic aim: was to assess the feasibility and acceptability of planning and delivering the intervention and trial methods to inform a full-scale non-inferiority trial. Outcome: the primary indicative efficacy outcome measures being field-tested are feasibility of participant recruitment and randomization, maintaining a protocol violation rate < 10%, and retaining 90% patient follow up 28 days after surgery. The primary clinical outcome measure of the future full “Tight K Study” will be incidence of AF after cardiac surgery.

**Discussion:**

The Tight K Pilot will assess the feasibility of conducting the full trial, which is intended to confirm or refute the efficacy of current potassium management in preventing AF after cardiac surgery.

**Trial registration:**

ClinicalTrials.gov, NCT03195647. Registered on 23 May 2017. Last updated 19June 2017.

**Electronic supplementary material:**

The online version of this article (doi:10.1186/s13063-017-2349-x) contains supplementary material, which is available to authorized users.

## Background

Approximately one in three patients is affected by atrial fibrillation (AF) after cardiac surgery, with most episodes occurring in the first five postoperative days [1–3]. AF occurrence is associated with increased short-term and long-term mortality [3–6], intensive care unit (ICU) and hospital stay [7, 8], and cost of care [9]. Persistence of this association after adjustment for potential confounding factors suggests that it may be causal [10]. The incidence and associated costs of AF are expected to increase as the surgical population ages [11].

Potassium plays an important role in cardiac electrophysiology [12]. Serum concentrations ([K^+^]) are commonly low following cardiac surgery [13], and appear marginally lower in in non-surgical cohorts among those suffering atrial arrhythmias [14]. Despite an absence of proof that this association is causal, efforts to maintain serum [K^+^] in the “high-normal” range (4.5–5.5 mEq/L), as opposed to just intervening if potassium drops below its lower “normal” threshold, are considered “routine practice” for AF prevention in patients post-surgery in many centres across the world [15].

The efficacy of the practice of maintaining high-normal serum potassium levels remains unproven and data supporting it are extremely limited, being derived from observational rather than interventional studies [15]. Indeed, there are no data to demonstrate that maintaining a high-normal potassium level is beneficial in these circumstances, or that aggressive replenishment of potassium in these patients improves outcome [16]. Meanwhile, potassium supplementation may cause discomfort or harm. Routine central venous potassium administration in the early post-operative period, when oral supplementation is not possible, is time-consuming, costly, and associated with clinical risk: rapid infusion can prove fatal [17], and leaving central venous catheters *in situ* for the sole purpose of potassium replacement increases infection risk. The annual costs of intravenous potassium exceed those for other drugs in many cardiothoracic units due to the large quantities administered [18]. Nursing time (e.g. for drug checks and administration) will also add to this cost. Oral potassium supplementation is commonly associated with gastrointestinal side effects and is often poorly tolerated by patients [19].

We here describe a trial (Tight K Pilot) designed to assess the feasibility of performing a randomized controlled non-inferiority trial to assess any impact of targeted maintenance of serum K+ concentration ≥ 3.6 mEq/L vs ≥ 4.5 mEq/L on AF incidence after coronary artery bypass grafting (CABG).

## Methods/design

The trial is a randomised feasibility trial. This protocol was written following the Standard Protocol Items: Recommendations for Interventional trials (SPIRIT) checklist (see Additional file 1).

Hypothesis for the pilot trial: it will be feasible to recruit and randomise 160 patients over a period of 6 months, maintain < 10% protocol violation rate, and retain 90% of patients for follow up 28 days post-surgery. The protocol violation rates are defined subsequently (“Endpoints of the Trial”).

Hypothesis for the main trial: AF will be no more common (based on a non-inferiority margin of 10%, see following) after cardiac surgery when serum potassium levels are maintained at ≥3.6 mEq/L (“relaxed control”) than when they are maintained at ≥4.5 mEq/L (“tight control”).

### Setting

The Tight K Pilot will be conducted in two UK centres – the Barts Heart Centre, Barts Health National Health Service (NHS) Trust and St George’s University Hospitals NHS Trust, both in London, UK.

### Trial population

Eligible patients are those undergoing elective CABG surgery.

### Exclusion criteria

1. Age < 18 years
2. Previous AF
3. Concurrent patient involvement in another clinical trial assessing post-operative interventions
4. Ongoing infection/sepsis at the time of surgery
5. Pre-operative high-degree atrio-ventricular (AV) block
6. Pre-operative serum potassium (K^+^) > 5.5 mEq/L
7. Current or previous use of medication for the purposes of cardiac rhythm management
8. Dialysis-dependent end-stage renal failure
9. Unable to give informed consent

### Informed consent procedure

Eligible participants will be given a copy of the patient information sheet (PIS) at a pre-operative hospital appointment or upon admission prior to surgery, at which time a delegated member of the research team will be available to discuss the trial further and to answer any questions that the patient may have. Research staff may approach patients prior to their scheduled hospital appointment via post, telephone or email to discuss the study.

All participants will be given at least 24 hours to consider whether or not to take part in the trial. If willing to take part, they can consent at any time prior to surgery. Written consent will be obtained on a consent form.

### Randomisation

One hundred and sixty eligible participants with informed written consent will be allocated in a 1:1 ratio using an online database (https://sealedenvelope.com/) to receive either tight (K^+^ ≥4.5mMol/L) or relaxed (K^+^ ≥3.6mMol/l) potassium control. Patients will be randomised on the day of surgery. The allocation will be stratified by site.

### Trial treatment period

**Fig. 1:**
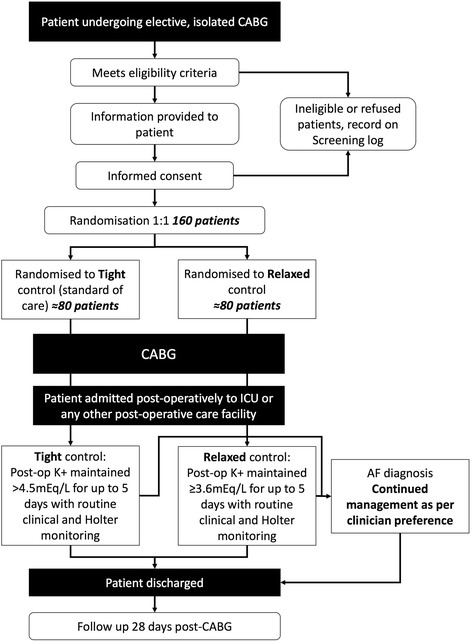
Flowchart of the study protocol. CABG, coronary artery bypass grafting; AF atrial fibrillation; post-op, post-operative

A flowchart of the trial treatment intervention process is shown in Fig. 1. The trial treatment intervention period will commence when the patient is admitted to ICU or any other post-operative care facility after surgery, according to local practice. It will end 120 hours (5 days) after that time point, or with occurrence of a clinically identified episode of AF (see following) - whichever occurs first.

### Tight potassium control

Patients randomised to the tight-control group will receive potassium supplementation if their serum K^+^ falls below 4.5 mEq/L (current practice).

### Relaxed potassium control

Those randomised to the relaxed-control group will receive potassium supplementation only if their serum K^+^ level drops below or equals 3.6 mEq/L.

### Potassium supplementation

The administration route used for all potassium replacement will be prescribed according to clinician preference and given according to existing standardised protocols. This supplementation may include intravenous (iv) or oral potassium formulation, administration of potassium-rich nasogastric feeding regimens or recommending the consumption of potassium-rich foods.

### Routine clinical practice

All other clinical practice (including the use of magnesium supplementation, the use of beta-blockers or anti-arrhythmic agents, the route of potassium administration, and blood tests) will be routine, and independent of trial allocation. In particular, the frequency with which serum [K^+^] is monitored will be according to existing protocols and clinician/nursing staff preference (Fig. 1).

### Patients with AF

**Fig. 2:**
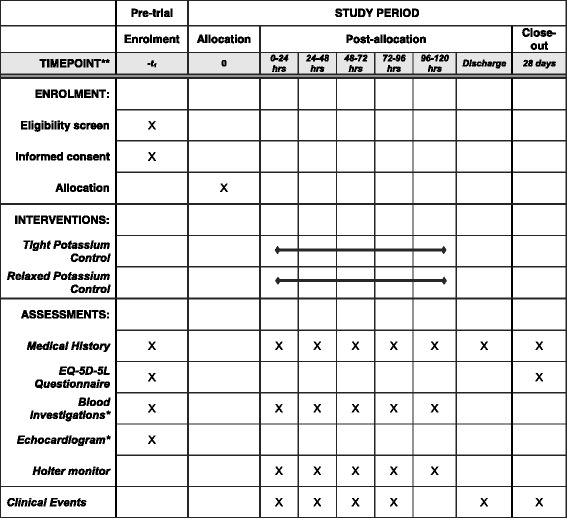
Standard Protocol Items: Recommendation for Interventional Trials (SPIRIT) figure depicting the schedule of enrolment, interventions and assessment. EQ-5D-5L, 5-level Euroqol-5D questionnaire

In keeping with recognised international criteria, atrial fibrillation will be defined as an episode of AF lasting ≥ 30 seconds that is clinically detected and/or electrocardiographically confirmed (on either a 12-lead electrocardiogram (ECG) or telemetry) [20]. Routine clinical monitoring will be supplemented by continuous Holter monitoring (eMotion Faros 180, Technomed Ltd) for the first 120 postoperative hours in all participants. Once a patient has a clinically identified period of AF, the trial treatment period ends and then there will be no restriction on potassium supplementation and they should be treated according to current practice (Fig. 1). The schedule of trial enrolment, interventions and assessments is shown in Fig. 2 (SPIRIT figure).

### Endpoints of the trial

The primary outcome measure of this pilot trial is the performance on feasibility endpoints.

Primary questions for the pilot trial are to investigate:

1. Whether it is feasible to recruit 160 patients over a period of 6 months (20% of those eligible)
2. Whether it is possible to randomise patients into groups for potassium replacement if K+ <4.5 mEq/L (usual practice) vs. < 3.6 mEq/L (lower limit of the normal range)
3. Whether it is feasible for the protocol violation rate to be no more than 10% (see following)
4. Whether it is feasible to maintain follow-up rates > 90% at 28 days after CABG

Reasons for a protocol violation may include:

1. A patient from the relaxed-control group being treated as if they are in the tight-control group or a patient from the tight-control group being treated as if they are in the relaxed-control group
2. Failure of randomisation
3. Alteration in planned surgery
4. Failure of the Holter monitoring process
5. Lack of data completion

Secondary endpoints for the pilot trial: in order to further inform the proposed main randomised controlled trial endpoints, the following additional outcomes will be collected:

1. Incidence and total duration of new-onset AF arrhythmia post-surgery until day 5. This is the primary endpoint for the future full Tight K Trial.
2. Mean critical care duration of stay.
3. Mean hospital duration of stay.
4. Incidence and total duration of all other arrhythmias until day 5 (120 hours), defined using standard diagnostic criteria.
5. Incidence of in-patient mortality.
6. Incidence of 28-day mortality.
7. Cost-effectiveness.

Holter recordings will be analysed after day 5 by clinical staff blinded to group allocation. The time of all clinically identiifed episodes of AF will be documented and contemporaneous Holter monitoring recordings will be reviewed to confirm or deny the AF diagnosis after day 5 by clinical staff blinded to treatment allocation. Cardiac arrhythmias that were not detected clinically will also be documented. A copy of all Holter reports will be sent to the patient’s primary admitting surgical team.

#### Health-related quality of life

All participants will complete a quality of life questionnaire (5-level Euroqol-5D (EQ-5D-5 L)) prior to their surgery and also at 28-day follow up, by telephone or post.

#### Post-operative follow up at 28 days

Patients surviving to hospital discharge will be followed up by telephone or post 28 days after randomization, to determine mortality and further episodes of heart rhythm problems (if known).

### Data collection

The data collected will be utilized to fulfill two distinct purposes:

1. To assess whether the primary and secondary endpoints of this study are met.
2. To assess the efficiency of data completion to ensure protocol compliance. To do this, the same variables that we intend to collate in the full future study will be collected in this pilot study.

The following will be collated over the trial study period (6 months):

- Total number of patients undergoing isolated CABG
- Number of patients screened, invited to participate, and recruited into the study.
- Patients not followed up at 28 days
- Number of protocol cross-overs and details
  - Patients in the relaxed-control group who inappropriately received potassium supplementation
  - Patients in the tight-control group who inappropriately did not receive potassium supplementation

For each protocol violation, a root-cause analysis will be undertaken that will examine staff roles, timing, education, and other preventable factors. All patients will also have a full medical history taken and various clinical examinations as part of usual care. The following will be recorded:

- Patient initials
- Ethnic origin
- Cardiac medication and indication (including beta-blockers, calcium channel blockers, ACE-inhibitors, angiotensin receptor blockers (ARBs), aldosterone receptor antagonists, anticoagulation)
- Medical history: chronic obstructive pulmonary disease (COPD)/lung disease, diabetes mellitus (and type), hypertension, myocardial infarction (MI), chronic kidney disease (CKD), transient ischaemic attack (TIA) or stroke/cerebrovascular accident (CVA), and family history of arrhythmia (and details)
- Imaging data: left ventricular ejection fraction (LVEF)/left atrial (LA) size, mitral regurgitation or stenosis (defined as moderate or worse)
- HAS-BLED and CHADSVASC scores will be calculated for patients at baseline

For each 24-hour study period (1 through 5), recorded serum potassium concentrations and potassium administration (dose and route) will be documented. Medication at hospital discharge will be collated, including whether anticoagulation is commenced for atrial fibrillation. Duration of stay on ICU, total hospital stay and mortality will be recorded. Adverse events attributed to K^+^ replacement, including gastrointestinal symptoms from oral K^+^ replacement will be collated. Detailed information will be collected on the resource use associated with delivering each protocol, including the total number of potassium replacement interventions.

### Statistical analysis

#### Power and sample size

One hundred and sixty patients are to be recruited from two centres, and randomly allocated in a ratio of 1:1. As this is a pilot trial to assess feasibility, power calculations are not appropriate. If the anticipated recruitment, follow-up and retention rates are demonstrated over a 6-month recruitment period, this would confirm the feasibility of a multicentre randomised controlled non-inferiority trial in 1682 patients with an estimated eligible cohort of 8700 patients over 3.5 years.

The sample size calculation for the main trial is based on a baseline incidence of AF of 35% [1, 21]; a clinically relevant non-inferiority margin of 10%; a true difference in favour of tight potassium control of 2%, 90% power; a one-sided alpha of 2.5% (equivalent to a two-sided alpha of 5%) and < 10% protocol violations. Due to this anticipated protocol violation rate, a greater number of patients will be recruited to the full study, as not all will be included in the final analysis.

#### Outcome analyses

The primary outcome measure of this pilot trial will be feasibility in terms of numbers recruited and randomised, protocol fidelity, and follow-up rates by trial arm. These statistics will inform a “Consolidated Standards of Reporting Trials” (CONSORT) diagram reporting recruitment, treatment, and retention.

Additionally descriptive summaries of baseline and follow-up data by arm will be tabulated. No significance tests will be performed to test for differences at baseline, or given that this is a pilot trial, at follow up. Descriptive statistics for continuous variables will include the mean, standard deviation, median, range, and the number of observations. Categorical variables will be presented as numbers and percentages. Exploratory analysis of the main trial outcomes will be by intention to treat (ITT) and given the non-inferiority trial design for the main trial, a per-protocol analysis will also be considered [22]. As this is a pilot trial, no interpretation will be made of any effect sizes and findings will primarily be used to help refine the design of the main trial. This will include assessment of rates of missing data. No formal analysis to account for missing data will be undertaken in this pilot trial. A detailed cost-effectiveness analysis will not be undertaken in this pilot trial, but simple analyses of cost-effectiveness will be performed utilizing data relating to data on quality of life and length of stay. Statistical analysis will be carried out blinded to treatment allocation.

### Ethical issues

The trial has been reviewed and ethically approved by the National Research Ethics Service London Committee – Camden and Kings Cross. The REC number is LO/17/0318. The trial will conform to the spirit and the letter of the declaration of Helsinki, and will be in accordance with the Barts Health NHS Trust and London School of Hygiene & Tropical Medicine (LSHTM) Good Clinical Practice (GCP) guidelines. The trial will be carried out in accordance with the ethical principles in the *Research Governance Framework for Health and Social Care, Second Edition*, 2005 and its subsequent amendments as applicable and applicable legal and regulatory requirements.

Confidentiality: participant data will be kept confidential and managed in accordance with the Data Protection Act, NHS Caldecott principles, The Research Governance Framework for Health and Social Care, and the conditions of Research Ethics Committee Approval.

Withdrawal of participants: a patient may decide to withdraw from the trial at any time without prejudice to their future care and will undergo standard clinical care. Patients will be encouraged to allow data and samples that have been collected before withdrawal to be used in the analyses. However, if consent to use data is also withdrawn, then these will be discarded. Patients withdrawing from the trial will continue to be followed up by their local clinical team. There should be no need for further follow up from the research team. The Clinical Trials Unit at LSHTM should be informed by email if a patient has withdrawn from the trial. A withdrawal form will be completed on the trial electronic case report form.

Safety reporting: Barts NHS Trust, as Sponsor of this trial, has responsibility to ensure arrangements are in place to record, notify, assess, report, analyse, and manage adverse events in order to comply with the UK regulations of Medicines for Human Use (Clinical Trials) Regulations 2004. All sites involved in the trial are expected to inform the Chief Investigator of any serious adverse events/reactions within 24 hours so that appropriate safety reporting procedures can be followed by the Sponsor.

All adverse events judged by either the investigator or the Sponsor as having a reasonable suspected causal relationship with potassium supplementation qualify as adverse reactions. Whilst any suspected, unexpected, serious adverse reaction (SUSAR) involving potassium will be reported according to the timelines for SUSARs, expected side effects of potassium will be reported in the end-of-trial safety report unless serious enough to warrant expedited reporting.

### Trial governance

Trial documentation: relevant trial documentation will be kept for a minimum of 15 years. Electronic data will be stored in a fully audited data centre in the UK with appropriate certifications including ISO 27001:2005 (Information Security) and 9001:2008 (Quality Management).

Trial registration and conduct: the trial is registered with ClinicalTrials.gov, identifier: NCT03195647. We will follow the Medical Research Council Guidelines on GCP in Clinical Trials. All investigators have been trained in GCP.

The Sponsor is Barts Health NHS Trust.

Trial Steering Committee (TSC): the trial will be overseen by the TSC, including an independent chair and at least two other independent members. The TSC will meet periodically during the trial.

Data Safety and Monitoring Committee (DSMC): the DSMC will be independent of the investigators and of the TSC, but will report to the TSC and (via the TSC to the Sponsor). The DSMC will consist of an independent chair, a senior statistician and at least one other senior clinician independent of the investigators. The DSMC will meet prior to the start of the trial and as this is a pilot trial their main role will be to monitor safety on an ongoing basis. They will also monitor data for quality and completeness. This will be facilitated through the development of a trial-specific database and an adverse-event database.

Trial management: the trial will be directed by the chief investigator and a project management group which will include the trial manager, data manager and trial statistician.

## Discussion

Disappointingly little progress has been made in effectively preventing AF after cardiac surgery [23]. Practice is diverse, and the supporting evidence base is weak [9]. Anecdotally, the commonest practice is to maintain serum [K^+^] in the high-normal range. The evidence base underpinning such practice is limited. Whilst postoperative potassium supplementation has been associated with reduced AF risk after CBP [2], such data are retrospective and no specific potassium target is suggested.

We have opted to address the efficacy of this practice in participants undergoing open CBP - a group that represents a large proportion of the total cardiac surgical workload. By choosing a large and comparatively homogenous patient cohort, the feasibility of delivering this study should be maximized.

A study that aims to conclusively assess the effectiveness of an established practice requires a non-inferiority, individually randomised design. In our case, that will require large patient numbers. It was felt important by both investigators and funders to perform a feasibility study before embarking on a large-scale trial in order to determine possible barriers to successfully implementing a seemingly simple protocol such as ours.

The investigators felt that it was important that any such study should be pragmatic and reflect “real-world” practice as far as possible. Therefore, minimal restrictions have been placed on the management of participants following surgery. Specifically, use of magnesium supplementation or antiarrhythmic drugs is unrestricted.

The true incidence of AF following cardiac surgery is controversial with a range of AF incidence reported in the literature, ranging from 10 to 65% [1]. The consensus from our Protocol Development Group was that our chosen non-inferiority margin of 10% is reasonable and pragmatic. Opting to utilize a smaller non-inferiority margin may make the future full study unviable, due to a significant increase in sample size and costs.

The findings of the future full study are intended to make a significant contribution to optimization of care for cardiac surgical participants. If effective, potassium supplementation to a high target level could be implemented as a standard of care, and costs related to peri-operative morbidity reduced. If potassium supplement is shown to have no benefit, the costs and risks of administration of potassium supplement that are avoided could be substantial.

### Trial status

The trial is currently being set up at two sites. Recruitment is scheduled to start at the first site by 1 August 2017. The proposed start date is 1 August 2017. The proposed end date is 28 February 2017 (end of follow up).

## Additional file

Additional file 1: Checklist of the Standard Protocol Items: Recommendations for Interventional Trials (SPIRIT) guidelines. (DOC 119 kb)

## Abbreviations

AF: Atrial fibrillation
CABG: Coronary artery bypass grafting
CONSORT: Consolidated Standards of Reporting Trials
ECG: Electrocardiogram EQ-5D-5 L, 5-Level Euroqol-5D
GCP: Good Clinical Practice
ICU: Intensive care unit
K^+^: Potassium
LSHTM: London School of Hygiene & Tropical Medicine
NHS: National Health Service
SPIRIT: standard protocol items: recommendation for interventional trials
SUSAR: Serious adverse reaction
